# Males of *Aedes aegypti* show different clock gene expression profiles in the presence of conspecific females

**DOI:** 10.1186/s13071-022-05529-8

**Published:** 2022-10-18

**Authors:** Jéssica Rodrigues Assunção Bezerra, Rafaela Vieira Bruno, Luciana Ordunha Araripe

**Affiliations:** 1 Laboratório de Biologia Molecular de Insetos, Instituto Oswaldo Cruz, Fundação Oswaldo Cruz, Rio de Janeiro, Brazil; 2 Instituto Nacional de Ciência E Tecnologia Em Entomologia Molecular (INCT-EM)/CNPq, Rio de Janeiro, Brazil

**Keywords:** *Aedes aegypti*, Social interaction, Reproductive behavior, Clock genes, Circadian gene expression, *cryptochrome 2*

## Abstract

**Background:**

The study of behavioral and physiological traits in mosquitoes has been mainly focused on females since males are not hematophagous and thus do not transfer the parasites that cause diseases in human populations. However, the performance of male mosquitoes is key for the expansion of populations and the perpetuation of mosquito species. Pre-copulatory communication between males and females is the initial and essential step for the success of copulation and studying the male facet of this interaction provides fertile ground for the improvement of vector control strategies. Like in most animals, reproduction, feeding, and oviposition are closely associated with locomotor activity in mosquitoes. Rhythmic cycles of locomotor activity have been previously described in *Aedes aegypti*, and in females, they are known to be altered by blood-feeding and arbovirus infection. In previous work, we found that males in the presence of females significantly change their locomotor activity profiles, with a shift in the phase of the activity peak. Here, we investigated whether this shift is associated with changes in the expression level of three central circadian clock genes.

**Methods:**

Real-time PCR reactions were performed for the gene *period*, *cycle*, and *cryptochrome 2* in samples of heads, antennae, and abdominal tips of solitary males and males in the presence of females. Assays with antennae-ablated males were also performed, asking whether this is an essential organ mediating the communication and the variation in activity profiles.

**Results:**

The gene *period* showed a conserved expression pattern in all tissues and conditions, while the other two genes varied according to the male condition. A remarking pattern was observed in *cry2*, where the difference between the amplitude of expression at the beginning of photophase and the expression peak in the scotophase was greater when males were in the presence of females. Antennae ablation in males did not have a significant effect on the expression profiles, suggesting that female recognition may involve other senses besides hearing and olfaction.

**Conclusion:**

Our results suggest that the expression of gene *cryptochrome 2* varies in association with the interaction between males and females.

**Graphical abstract:**

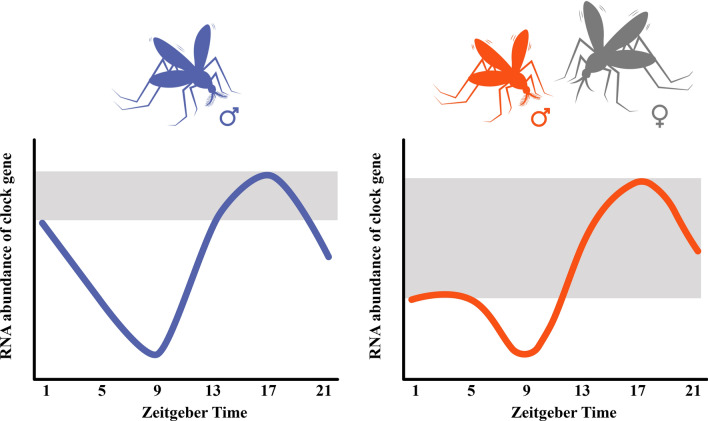

**Supplementary Information:**

The online version contains supplementary material available at 10.1186/s13071-022-05529-8.

## Background

*Aedes aegypti* (Diptera: Culicidae) mosquitoes are the main vectors of arboviruses such as dengue, Zika, chikungunya, and urban yellow fever viruses [1]. Biological traits and environmental factors contribute to the maintenance and expansion of *Ae. aegypti* populations, favoring the risk of epidemic diseases. Therefore, it is important to study the behavioral and physiological traits that account for the rise in mosquito populations to mitigate these risks. One particularity of mosquito biology is that only females can blood seek and bite, given that a blood meal is necessary for completing ovogenesis but not required for survival [2]. Thus, most research focuses on females, the actual pathogen vectors, and studies focusing on males are still incipient [3–6]. However, the expansion of mosquito populations depends on reproductive success, and understanding male reproductive behavior and physiology is crucial for the framework of any program of vector control. Pre-copulatory communication is an important initial step to recognition and courtship among the events contributing to reproductive success. Thus, a failure in the recognition between males and females can restrain the growth of mosquito populations.

Behaviors such as reproduction, foraging, feeding, and oviposition are closely associated with the locomotor activity. Like most organisms, mosquitoes exhibit rhythmic cycles of locomotor activity, which are regulated by an endogenous pacemaker called the circadian clock [7]. In the model species *Drosophila melanogaster*, the circadian clock works through genes that interact in negative feedback loops and determines that physiological and behavioral processes occur in approximately 24 h. This pattern of regulation has been found in a variety of other organisms, from microbes to mammals [8, 9], being considered a conserved characteristic of the endogenous clock. Environmental factors such as light and temperature synchronize the circadian clock in cycles of exact 24 h [10].

In *D. melanogaster*, the central clock is located in the brain and is composed of about 150 neurons that express clock genes [11]. In addition, peripheral clocks in other organs also express the clock genes and may exhibit rhythmic patterns. These peripheral clocks can function independently of the central clock, regulating specific functions of each organ [11–14], and were described in various tissues in *Drosophila* and other organisms, such as in reproductive organs, antennae, Malpighian tubules, and others [15–20]. In a critical study, using transgenic *Drosophila* expressing the construct *per-luc* (the clock gene *period* fused to the luciferase gene—*luc*) or *per*-driven GFP (*period*-driven green fluorescent protein), the authors analyzed the circadian oscillations in different tissues [16]. Through experiments where different organs were dissected from transgenic flies and subsequently incubated, it was found that heads, thoraxes, abdomens, and also small organs like antennae and proboscis exhibited rhythmic oscillations. The results suggest that individual cells can keep their circadian oscillations even in the absence of a brain [16].

Previous efforts have been made to elucidate the molecular bases of the circadian clock of *Ae. aegypti* mosquitoes. Our group described the RNA expression profiles of the main circadian clock genes in females of this species [21]. The results revealed remarkable contrast with *D. melanogaster* profiles: for instance, unlike the arrhythmic pattern in the fly [22], the gene *cyc* showed rhythmic expression in *Ae. aegypti* females [21]. Furthermore, while *D. melanogaster*'s genome has one *cryptochrome* gene with photoreceptor function, two *cryptochrome* genes were found and analyzed in *Ae. aegypti*: *cry1*, orthologous to *Drosophila*’s *cry*, and *cry2*, a plausibly transcriptional repressor also found in butterflies, beetles, and bees [21, 23, 24]. Only *cry2* showed a rhythmic expression profile, exhibiting a bimodal pattern with two expression peaks: one at the early hours of the photophase and the second peak in the scotophase [21].

Besides varying among organisms, rhythmic patterns of locomotor activity are not steady within a given species. Factors that influence the physiological state of organisms may lead to alterations in locomotor activity profiles, as described in female *Ae. aegypti* after blood-feeding [25] and after arboviruses infection [26, 27]. Intraspecific interactions may also bring in locomotor activity variation, as shown in laboratory experiments with male *Ae. aegypti* in the presence of females [28]. In this previous study, we analyzed the locomotor/flight activity over 24 h of solitary males and males exposed to females at a perceptible distance. Males exposed to females had their locomotor/flight activity patterns significantly altered compared to solitary males: at dusk, their second activity peak was significantly greater than the second peak of solitary males. Also, this second activity peak overlaps with the single peak of females, suggesting that males match their activity peak with the phase in which females are most active. We conducted experiments using individuals with ablated antennae or ablated wings to understand which signals could change males' activity patterns when exposed to females. The results suggested that males perceive females even when females are wingless and do not produce the wing-beat sounds, indicating that signals other than wing-beat sounds may be involved in recognition between males and females, possibly chemical and/or visual cues [28]. Evidence of sexual pheromones, although still uncertain for *Ae. aegypti*, was seen in other dipterans like *Lutzomyia longipalpis* [29, 30].

Whether alterations in rhythmic behavior are backed by changes in the cycling pattern of clock genes’ expression is a relevant question, especially in the frame of reproductive behavior and its significance for the expansion of *Ae. aegypti* populations. Here, we describe the cycling of three of the main clock genes in tissues of male *Ae. aegypti*, in and out of female presence, and associate the gene expression profiles with the previous work's locomotor/flight activity profiles. Furthermore, we investigate the role of the male antennae, the main hearing and olfaction organ in mosquitoes, in perceiving the female conspecific and mediating the variation in gene expression cycling.

The gene *period* was the first clock gene described and has been shown to carry several polymorphisms associated with reproductive traits, for instance the mating activity [31] and copulation duration in *Drosophila* [32], and to be an important molecular marker for taxonomy and evolutionary studies of sympatric species in *Drosophila* [33] and in the dipteran *Lutzomyia longipalpis* [34–36]. The gene *cycle*, while constitutively expressed in *Drosophila* [22], shows rhythmic expression in *Ae. aegypti* [21]. Also, it is known that CYC has an important role in the regulation of output genes [37]. The gene *cry2*, not found in *Drosophila*, has been cited as a gene with variable expression in previous literature on mosquitoes and other organisms [21] and has been indicated to be a transcriptional repressor acting on the regulation of reproductive behavior in a species of moth [38].

## Methods

### Mosquitoes

The experiments were carried out with *Ae. aegypti* mosquitoes (Rockefeller strain) provided by the Laboratory of Physiology and Control of Arthropod Vectors (Fiocruz, Brazil). Eggs were hatched in plastic containers with 1.5 l Milli-RO water and 1 g yeast (Vitalab^®^, Brazil) as a food source. The larvae were redistributed to 300 larvae per container at the first instar. The water was changed, and 1 g of yeast was added every 2 days until the pupation stage [39]. The pupae were counted, collected, and separated in cages with 10% sucrose solution ad libitum. After emergence, the virgin females and males were collected twice a day and placed in different cages until further use in experiments. Individuals from larval to adult stages were synchronized to 12 h of light and 12 h of darkness (LD12:12) under a constant temperature of 25 °C. All individuals entered the experimental tubes at 3–5 days of age.

### Experimental design

To recreate the same conditions used in our previous work, where individual males’ locomotor/flight activity was monitored in or out of females’ presence [28], a confinement system set with pairs of transparent plastic tubes was created. The confinement system consisted of 15-ml Falcon^®^ tubes, whose caps were cut through in their center for the adaptation of a tulle net. Males and females were placed individually inside the tubes, and pairs of tubes were tape-attached, with their caps facing each other. While the tulle net allows males and females to perceive each other’s presence, it prevents copulation. A set of tubes was left empty and paired with tubes with males. Inside the tubes, a piece of cotton soaked in 10% sucrose solution was provided as a food source ad libitum. The experiments were performed in four conditions: “solitary males” and “males with females” with 50 pairs of tubes for each collection point, and “antennae-ablated solitary males” and “antennae-ablated males with females” with 20 pairs of tubes for each collection points.

Male mosquitoes with ablated antennae were used as an experiment condition because antennae are the main communication organs between males and females, acting in hearing and olfaction. Since the sound of wing beating during the flight is perceived by specific structures in the antennae [40, 41], the ablation of males' antennae can interfere with the communication required for the encounter and copulation. For antennae ablation, males were anesthetized on ice, and the organs were mechanically removed with a pair of tweezers. Males were placed in the experimental tubes before waking up and were observed for several minutes after waking up to assure they were alive and moving.

### Experimental conditions and samples

To study the expression of clock genes over 24 h, we maintained the mosquitoes in the paired tubes under LD12:12 and 25 °C over 4 days. The study of circadian gene expression requires a sampling schedule at regular intervals over 24 h. The collection and immediate freezing of mosquitoes every 4 h allow a reliable assembly of the expression profiles of circadian clock genes [42]. The samples were collected on the 4th day at six different *Zeitgeber* times (ZTs) (ZT1, ZT5, ZT9, ZT13, ZT17, and ZT21). The ZTs stand for the number of hours passed after lights turn on. Tubes were immersed in liquid nitrogen for males’ instant freeze. Males were then transferred to the surface of an ice-frozen metal plate and dissected under a stereoscopic microscope. The tissues of interest were heads, antennae, and abdominal tips (containing testes and accessory glands), which were separately stored in microcentrifuge tubes with 100 µl of TRIzol and maintained at − 80 °C for posterior RNA extraction. For each biological replicate, 50 pairs of antennae, 20 heads, and 20 abdominal tips were dissected from the males of each condition and ZT.

### RNA extraction, cDNA synthesis, and qPCR

The plumose antennae of male mosquitoes are composed of different structures that show varying degrees of sclerotization [43]. However, the tissue is mostly stiff, and standard RNA extraction protocols yield insignificant mass, even with an increased number of individuals sampled. After several trials, we developed a hybrid RNA extraction protocol that showed successful results, providing enough mass for all the replicates in every quantitative real-time PCR (qRT-PCR) experiment.

The hybrid protocol merges the TRIzol RNA extraction protocol (Invitrogen, Carlsbad, CA, USA) and the ReliaPrep^™^ RNA Tissue Miniprep System (Promega Corp., Madison, WI, USA). Briefly, the samples were homogenized in 100 µl TRIzol, and the volume was completed to 1000 µl with the same reagent. After that, 200 µl chloroform was added, the samples were centrifuged, and the aqueous phase was transferred to new microcentrifuge tubes. From this stage on, starting with the addition of 340 µl of isopropanol (Sigma-Aldrich), the ReliaPrep protocol was followed. The total RNA was quantified by Qubit Fluorometer Quantification (Invitrogen, Carlsbad, CA, USA), using the Qubit RNA HS Assay Kit, according to the manufacturer's protocol. After quantification, the samples were diluted to 5 ng/µl. The cDNA was synthesized with TaqMan Reverse Transcription Reagents (Applied Biosystems, Foster City, CA, USA), as described by the manufacturer’s protocol, and diluted to 1 ng/µl. The relative mRNA abundances of each condition, tissue, and collection times were determined by qRT-PCR using the Power SYBR Green PCR Master Mix (Applied Biosystems, Foster City, CA, USA) in a StepOnePlus^™^ Real-Time PCR System (Applied Biosystems). The analyses were performed for the clock genes *period*, *cycle*, and *cryptochrome 2*, using gene *rp49* as an endogenous control. The oligonucleotides of all genes used here were designed previously by Gentile et al. [21, 44] and fit the amplification efficiency criteria required for the analysis. We used the 2^−ΔΔCT^ method to analyze the data from three technical replicates for each sample [45].

### Statistical analysis

Statistical analyses were performed using GraphPad Prism version 5.0.2 (GraphPad Software, San Diego, CA, USA). The 2^−ΔΔCT^ values were compared among ZTs by ANOVA, followed by Tukey’s a posteriori test, where *P* < 0.05 was considered significant. The significance given by ANOVA indicates that gene expression varies among different collection points (ZTs). Graphic profiles of the gene expression variation were constructed with 2^−ΔΔCT^ values. Next, the software CircWave (version 1.4, University of Groningen, The Netherlands) was run using the 2^−ΔΔCT^ values. CircWave fits sinusoidal curves to the individual expression data and compares the fitting with a continuous line calculated through the data mean [46]. A significant difference between the fitted sinusoidal curve and the horizontal line, given by *P* < 0.05, was used to confirm the rhythmicity of expression.

## Results

Results are presented in sections, each describing the expression profiles of a gene in the three tissues and four conditions studied. The *P* values in parentheses stand for the significance of Tukey post hoc tests comparing high and low mRNA abundances (peaks and troughs) among ZTs. The average mRNA abundance values and standard errors for each collection point are presented in the Additional file 1: Table S1. Raw data sheets are provided as Additional files 2: Table S2 , 3: Table S3, 4: Table S4, 5: Table S5, and 6: Table S6.

### *period* expression profile in tissues of *Ae. aegypti* males

**Fig. 1:**
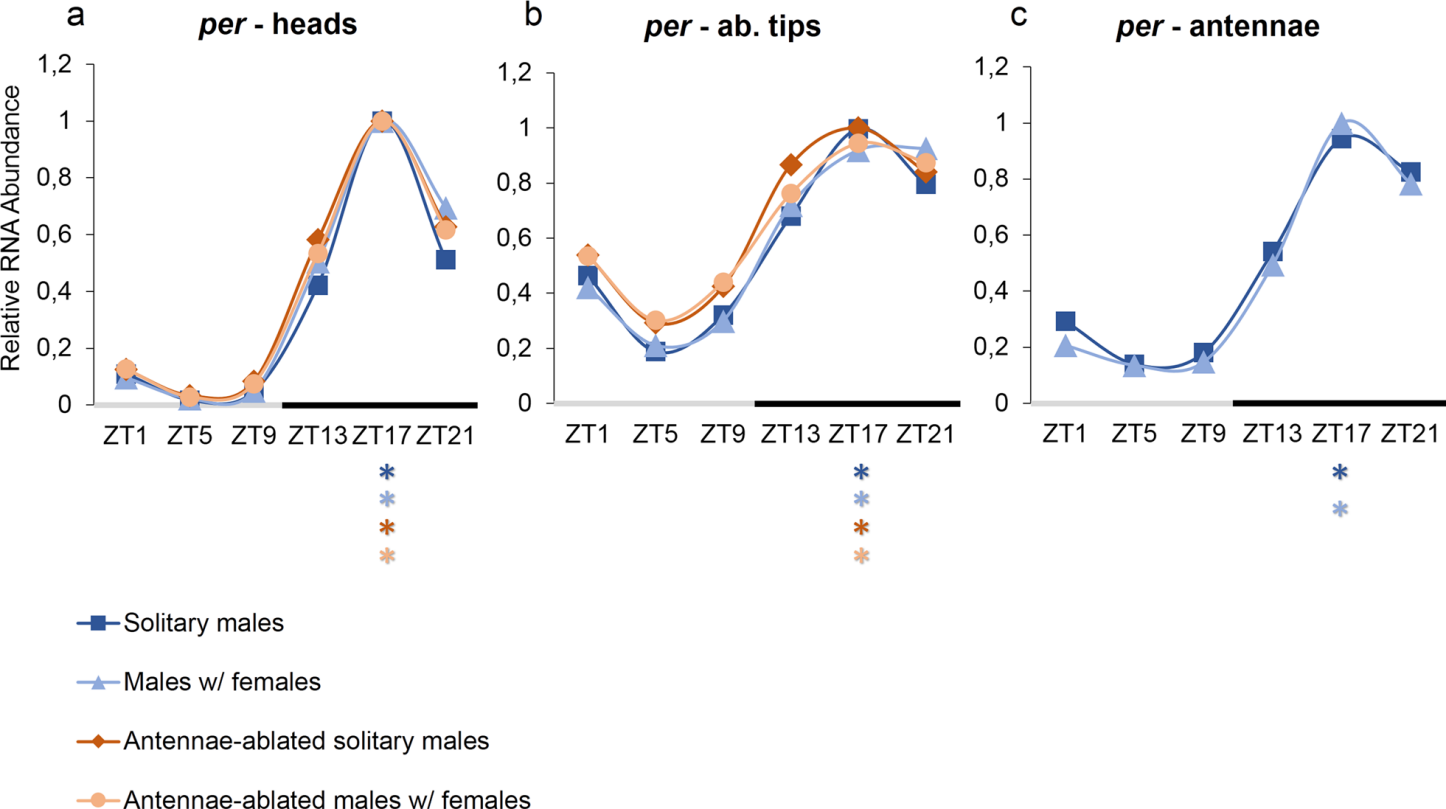
RNA expression profile of *per* gene, over 24 h (LD12:12), in tissues of *Aedes aegypti* male mosquitoes. Relative RNA abundance of *per* in (**a**) heads, (**b**) abdominal tips and (**c**) antennae of *Ae. aegypti* males. Gene expression was determined by quantitative real-time PCR, and graphs were generated from the average of three independent experiments. The y-axis shows the relative RNA abundance, and the x-axis shows the *Zeitgeber* time (ZT). The gray bar represents the light phase, and the black bar represents the dark phase. Asterisks below the x-axis represent expression differences among high and low values (peaks and troughs), according to Tukey’s a posteriori test. Standard errors are not displayed here for visual ease; instead, they are shown in Additional file 1: Table S1

The circadian expression profiles of *per* in heads, abdominal tips, and antennae are shown in Fig. 1. In all tissues and conditions, *per* showed expression varying significantly among ZTs (Table 1), with an expression peak at ZT17 (Tukey: *P* < 0.001), and rhythmicity was confirmed by the results of CircWave (Table 1). Within each tissue studied, the circadian profiles of the four conditions were akin.

**Table 1:** Statistical analysis of the gene* period* in tissues of *Ae. aegypti* males

Conditions	Gene: tissues					
	*per*—heads		*per*—ab. tips		*per*—antennae	
	ANOVA	Circwave	ANOVA	Circwave	ANOVA	Circwave
Solitary males	*F5,12* = 350.9	*R*^*2*^ = 0.99167	*F5,12* = 21.42	*R*^*2*^ = 0.85767	*F5,12* = 62.29	*R*^*2*^ = 0.9584
	*P* < 0.001	*P* < 0.001	*P* < 0.001	*P* < 0.001	*P* < 0.001	*P* < 0.001
Males w/females	*F5,12* = 138.5	*R*^*2*^ = 0.96809	*F5,12* = 28.36	*R*^*2*^ = *0.89218*	*F5,12* = 26.46	*R*^*2*^ = 0.89994
	*P* < 0.001	*P* < 0.001	*P* < 0.001	*P* < 0.001	*P* < 0.001	*P* < 0.001
Antennae-ablated solitary males	*F5,12* = 372.3	*R*^*2*^ = 0.99312	*F5,12* = 47.35	*R*^*2*^ = 0.95106		
	*P* < 0.001	*P* < 0.001	*P* < 0.001	*P* < 0.001		
Antennae-ablated males w/females	*F5,12* = 277.5	*R*^*2*^ = 0.99144	*F5,12* = 26.17	*R*^*2*^ = 0.8784		
	*P* < 0.001	*P* < 0.001	*P* < 0.001	*P* < 0.001		

### *cycle* expression profile in tissues of *Ae. aegypti* males

**Fig. 2:**
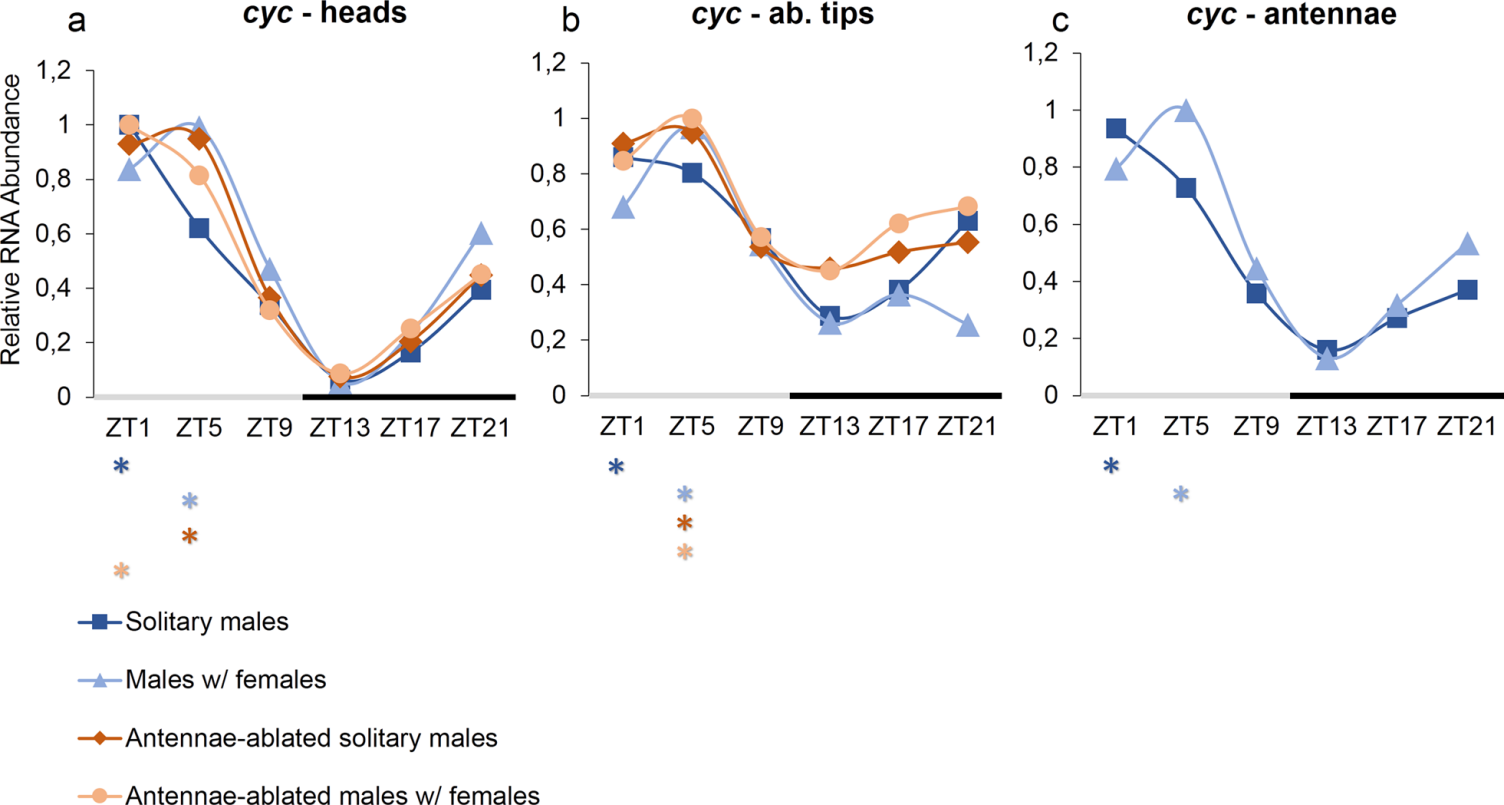
RNA expression profile of *cyc* gene, over 24 h (LD12:12), in tissues of *Aedes aegypti* male mosquitoes. Relative RNA abundance of *cyc* in (**a**) heads, (**b**) abdominal tips, and (**c**) antennae of *Ae. aegypti* males. Gene expression was determined by quantitative real-time PCR, and graphs were generated from the average of three independent experiments. The y-axis shows the relative RNA abundance, and the x-axis shows the *Zeitgeber* time (ZT). The gray bar represents the light phase, and the black bar represents the dark phase. Asterisks below the x-axis represent expression differences among high and low values (peaks and troughs), according to Tukey’s a posteriori test. Standard errors are not displayed here for visual ease; instead, they are shown in Additional file 1: Table S1

The circadian expression profiles of *cyc* in heads, abdominal tips, and antennae are shown in Fig. 2. In different tissues and conditions, the gene *cyc* showed an expression peak oscillating between ZT1 and ZT5 and a conserved trough at ZT13. In heads, *cyc* reached an expression peak at ZT1 in the conditions “solitary males” (*P* < 0.001) and “antennae-ablated males with females” (*P*< 0.001) (Fig. 2a). In the conditions “males with females” (*P*< 0.001) and “antennae-ablated solitary males” (*P*< 0.001), the expression peak was reached at ZT5 (Fig. 2a). In abdominal tips, the gene *cyc* showed an expression peak at ZT1 in the condition “solitary males” (*P* < 0.05) and at ZT5 in the conditions “males with females” (*P*< 0.001), “antennae-ablated solitary males” (*P* < 0.001) and “antennae-ablated males with females” (*P* < 0.01) (Fig. 2b). In antennae, *cyc* expression reached a peak at ZT1 in the condition “solitary males” (*P* < 0.001) and a peak at ZT5 in the condition “males with females” (*P*< 0.001) (Fig. 2c). ANOVA showed a significant difference among ZTs in all conditions, and CircWave confirmed the rhythmic profile of *cyc* (Table 2).

**Table 2:** Statistical analysis of the gene *cycle* in tissues of *Aedes aegypti* males

Conditions	Gene: tissues					
	*cyc*—heads		*cyc*—ab. tips		*cyc*—antennae	
	ANOVA	Circwave	ANOVA	Circwave	ANOVA	Circwave
Solitary males	*F5,12* = 88.61	*R*^*2*^ = 0.88609	*F5,12* = 4.107	*R*^*2*^ = 0.60175	*F5,12* = 43.69	*R*^*2*^ = 0.88426
	*P* < 0.001	*P* < 0.001	*P* < 0.05	*P* < 0.01	*P* < 0.001	*P* < 0.001
Males w/females	*F5,12* = 23.64	*R*^*2*^ = 0.87511	*F5,12* = 13.58	*R*^*2*^ = 0.8028	*F5,12* = 15.66	*R*^*2*^ = 0.85931
	*P* < 0.001	*P* < 0.001	*P* < 0.001	*P* < 0.01	*P* < 0.001	*P* < 0.001
Antennae-ablated solitary males	*F5,12* = 145.3	*R*^*2*^ = 0.96299	*F5,12* = 15.05	*R*^*2*^ = 0.86086		
	*P* < 0.001	*P* < 0.001	*P* < 0.001	*P* < 0.001		
Antennae-ablated males w/females	*F5,12* = 187.0	*R*^*2*^ = 0.95009	*F5,12* = 7.870	*R*^*2*^ = 0.76567		
	*P* < 0.001	*P* < 0.001	*P* < 0.01	*P* < 0.001		

### *cryptochrome 2* expression profile in tissues of *Ae. aegypti* males

**Fig. 3:**
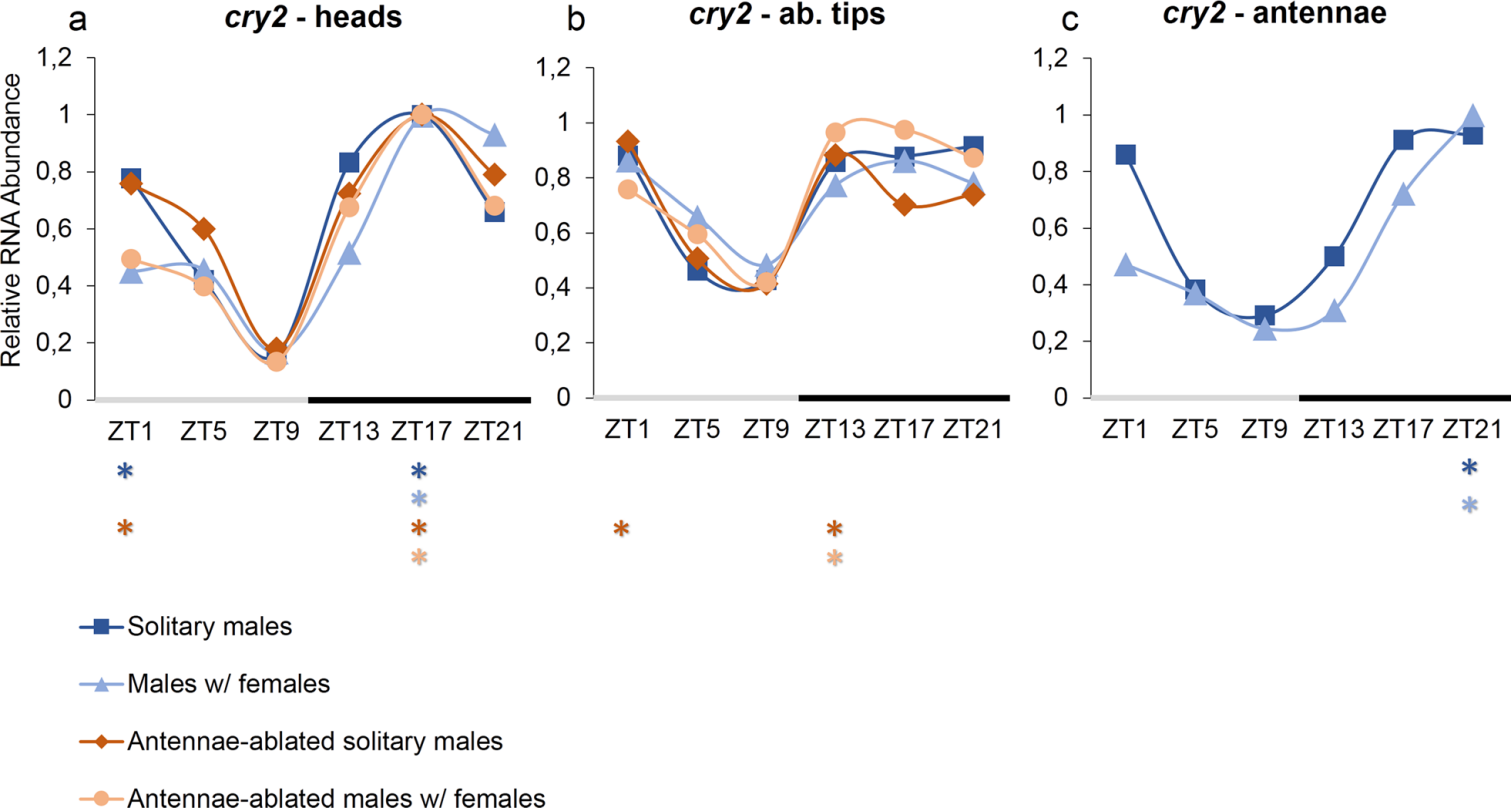
RNA expression profile of *cry2* gene, over 24 h (LD12:12), in tissues of *Aedes aegypti* male mosquitoes. Relative RNA abundance of *cry2* in (**a**) heads,  (**b**) abdominal tips, and (**c**) antennae of *Ae. aegypti* males. Gene expression was determined by quantitative real-time PCR, and graphs were generated from the average of three independent experiments. The y-axis shows the relative RNA abundance, and the x-axis shows the *Zeitgeber* time (ZT). The gray bar represents the light phase, and the black bar represents the dark phase. Asterisks below the x-axis represent expression differences among high and low values (peaks and troughs), according to Tukey’s a posteriori test. Standard errors are not displayed here for visual ease; instead, they are shown in Additional file 1: Table S1

The circadian expression profiles of *cry2* in heads, abdominal tips, and antennae are shown in Fig. 3. For this gene, expression profiles were more heterogeneous among tissues and conditions. In heads, *cry2* showed two expression peaks (ZT1 and ZT17) in the conditions “solitary males” (*P* < 0.001) and “antennae-ablated solitary males” (*P* < 0.001) (Figs. 3a). In the conditions “males with females” and “antennae-ablated males with females” *cry2* showed a single peak at ZT17 (*P*< 0.001) (Fig. 3a). In all conditions, ANOVA showed significant expression variation among ZTs, and CircWave confirmed the rhythmic profile of *cry2* (Table 3). A remarking and replicable difference in RNA abundances was found: in conditions where males were kept in the presence of females, the mRNA abundance of *cry2* at ZT1 was lower than that observed for solitary males (Fig. 3a). The relative values at ZT1 were compared between solitary males and males with females with a Student t-test, which confirmed significant difference in heads of antennae-ablated males (*t* = 4.360, *P* < 0.05) and in antennae (*t* = 3.074, *P* < 0.05). In heads of non-ablated males the same trend was observed, though with no significant difference (*t* = 2.157, *P*= 0.0972).

**Table 3:** Statistical analysis of the gene *cryptochrome 2* in tissues of *Ae. aegypti* males

Conditions	Gene: tissues					
	*cry2*—heads		*cry2*—ab. tips		*cry2*—antennae	
	ANOVA	Circwave	ANOVA	Circwave	ANOVA	Circwave
Solitary males	*F5,12* = 15.35	*R*^*2*^ = 0.84661	*F5,12* = 5.033	*R*^*2*^ = 0.53803	*F5,12* = 26.69	*R*^*2*^ = 0.87674
	*P* < 0.001	*P* < 0.001	*P* < 0.05	*P* < 0.01	*P* < 0.001	*P* < 0.001
Males w/females	*F5,12* = 4.190	*R*^*2*^ = 0.49139	*F5,12* = 1.647	*R*^*2*^ = 0	*F5,12* = 26.13	*R*^*2*^ = 0.90135
	*P* < 0.05	*P* < 0.01	*P* = 0,2217	*P* = NaN	*P* < 0.001	*P* < 0.001
Antennae-ablated solitary males	*F5,12* = 39.68	*R*^*2*^ = 0.87807	*F5,12* = 5.315	*R*^*2*^ = 0		
	*P* < 0.001	*P* < 0.001	*P* < 0.01	*P* = NaN		
Antennae-ablated males w/females	*F5,12* = 123.2	*R*^*2*^ = 0.91214	*F5,12* = 6.432	*R*^*2*^ = 0.4884		
	*P* < 0.001	*P* < 0.001	*P* < 0.01	*P* < 0.01		

*NaN* not a sinusoidal curve, arrhythmic expression

In peripheral tissues, the patterns were not as consistent among conditions. In abdominal tips, the relative mRNA abundance of *cry2* varied significantly for “solitary males” and the CircWave analysis confirmed rhythmicity (Table 3). In the condition “males with females” no significant differences between ZTs were found, and CircWave confirmed the arrhythmic profile (Table 3). Significant expression peaks at ZT1 (*P*< 0.05) and ZT13 (*P*< 0.05) were observed with ANOVA’s post-hoc Tukey in the condition “antennae-ablated solitary males” but CircWave revealed an arrhythmic profile (Table 3). In the condition “antennae-ablated males with females” *cry2* presented a single expression peak at ZT17 (*P* < 0.01) (Fig. 3b), and CircWave showed a low value of R^2^, though with significant rhythmicity (Table 3). In antennae, *cry2* showed a significant expression variation among ZTs, with rhythmicity confirmed by CircWave (Table 3). A profile with one single peak at ZT21 in the conditions “solitary males” (*P*< 0.001) and “males with females” (*P*< 0.001) was found (Fig. 3c). Notably, as well as in heads, the RNA abundance of *cry2* at ZT1 was relatively lower in “males with females” than in “solitary males” (Fig. 3c).

## Discussion

In a precursor study, we measured the locomotor/flight activity of *Ae. aegypti* males’ mosquitoes in response to confined females of the same species. Diverse conditions were studied, among them males in the presence of females and males in the presence of wingless females. The results revealed that the males could notice the females even in the absence of wing-beat sounds and align their activity patterns with those of females [28]. Given that, we hypothesized that the changes in the activity patterns of males exposed to females could result from a difference in the RNA expression of circadian clock genes. Thus, we herein investigated the RNA circadian expression of genes *period*, involved in the first loop of the circadian clock described; *cycle*, whose protein forms the heterodimer CLK-CYC and activates the main clock genes’ expression; and *cryptochrome 2*, which was suggested to have a role in the temporal activity pattern that distinguishes nocturnal and diurnal mosquitoes [21].

Several species exhibit rhythmic fluctuations of a variety of behavioral and physiological processes. In the model species *D. melanogaster*, previous studies have demonstrated the influence of clock genes on reproductive behavior [47]. In one important example in the literature [31], the authors demonstrated that the circadian clock strongly controls the mating activity of this species, and experiments using individuals carrying *per*^01^ mutation showed that their mating frequency was negatively affected [31]. In our results, the gene *per* showed a conserved expression profile in heads, antennae, and abdominal tips of *Ae. aegypti* males’ mosquitoes in all conditions studied, independently of females’ presence. We believe that, because *per* is involved in the central regulation of the circadian clock and showed consistent RNA expression profiles among tissues, its RNA expression may not be under the influence of social interaction represented by the proximity of a female, as studied here.

The expression profile of *cyc* in females’ heads was described by our group as exhibiting a rhythmic pattern, with a peak of RNA expression at ZT3 and a trough at ZT13 in the LD regime [21]. In the current study, the expression peak of *cyc* varied in males' tissues between ZT1 and ZT5 according to the condition studied. It is noticeable that when females are present with non-ablated males, *cyc* seemed to shift its expression peak to a later phase (from ZT1 to ZT5), as seen in Fig. 2. More specifically, in ZT5 the mRNA expression is higher in heads of males when females are present (*t* = 3.523, *P*< 0.05), but not in antennae-ablated males with females (*t* = 1.236, *P* = 0.284). The pattern of expression in antennae visually corroborates with the results in heads of non-ablated males (Fig. 2c), although the difference was not significant (*t* = 1.999. *P*= 0.1162). The similar profiles suggest that *cycle*’s expression may be influenced by the presence of females when antennae are present mediating the communication. In Gentile et al. [21], *cycle* expression was studied in female heads every 2 h, and they found the peak of expression at ZT3, which curiously is between the varying ZT1 and ZT5 we found. Interestingly, in every sample analyzed in our and Gentile’s works, the trough of expression was very conserved, at ZT13, which indicates that one gene’s circadian functionality may also be associated with the trough.

On the other hand, the gene *cry2* had its expression modified in male tissues depending on the condition studied. *cry2* is believed to function as a transcriptional repressor [23,24] and has been implicated in the regulation of insect behavior, including reproductive behavior [38]. The RNA expression profiles of *cry2* were described in female heads of *Ae. aegypti* and *Culex quinquefasciatus* by our group [21]. It was found that the pattern of expression differed among the species: while in *Cx. quinquefasciatus cry2* showed a single expression peak in the scotophase in *Ae. aegypti cry2* showed a bimodal profile, with an expression peak in the photophase and another peak in the scotophase. The authors argued that the difference between the two species might be related to their contrasting activity phases since *Ae. aegypti* has diurnal habits, and *Cx quinquefasciatus* is a nocturnal species [21].

Here, we found that *cry2* showed a bimodal profile in heads of solitary males, with and without antennae, but when males were exposed to females, *cry2* was much less expressed at ZT1, and the first peak collapsed (Fig. 3). Thus, males with females showed a single expression peak of *cry2* in heads. This intriguing effect may suggest that the first expression peak of *cry2* is influenced by the social interaction between males and females. In antennae, *cry2* cycled with only one expression peak in both conditions, and the reason for this could be that antennae are peripheral organs and may be under the regulation of peripheral clocks, presenting their own rhythmic oscillations [16]. In abdominal tips, although the rhythmicity of *cry2* expression was not statistically supported in all conditions, a graphical analysis suggests similar curves between rhythmic and arrhythmic profiles, which indicates a trend in daily patterns of expression variation.

Notably, the ablation of males' antennae, which are the organs responsible for olfactory and acoustic perception [41, 48], did not seem to affect the gene expression of *cry2* since the expression profiles were consistent between conditions that diverged solely by the presence of antennae (Fig. 3a). The most important factor associated with changes in *cry2* expression profile seems to be the social interaction given by the presence of females (Fig. 3a, c). In a previous study, we observed that antennae ablation dramatically reduces the amplitude of locomotor/flight activity of males and makes their activity profiles with and without females even [28]. This result is somewhat paradoxical: the antennae are considered the main perception organ in mosquitoes, and, in fact, our results show that males without antennae do not perceive females’ presence in a way that provokes a shift in their behavior [28]. However, changes are seen at the regulatory levels when females are present, with significantly different profiles of *cry2* expression. Therefore, in the absence of males’ antennae, the perception of confined females must be made possible by a sense other than hearing and olfaction, possibly through vision or touch (through the tulle net), without affecting locomotor/flight activity.

In addition, the fact that the main difference in *cry2* expression is a variation in the relative amplitude of RNA abundance at ZT1 (beginning of the photophase) suggests that the first expression peak may be more influenced by external factors. In fact, in another study of our group, analyses of *cry2* expression in *Ae. aegypti* female heads, submitted to different cycles of temperature and photoperiod, also showed variation in the amplitude of the morning peak of *cry2* [49]. The authors also argued that the first expression peak of *cry2* may have a function similar to *cry1* expression in the mating isolation of two sibling species of tephritid fruit flies [49, 50]. At the phenotypic level, males exposed to females also showed a lower amplitude in the first evening peak activity than in the second evening peak [28]. However, the association of the observed difference in *cry2* expression with the patterns observed in the locomotor/flight activity is still premature. A functional study of *cry2*, accompanied by observations of its effects on social interaction or female recognition, could help to elucidate whether the two phenomena are associated.

## Conclusions

The current study advances the understanding of clock genes' association with male mosquitoes' sensory perception of mates, a field where studies are still incipient. In fact, to our knowledge, this is a pioneer study of clock genes' expression in *Ae. aegypti* male tissues and in the context of reproductive behavior. Here, we explored the social interaction related to recognition for mating, given by the perception of females by males, and its effects on the RNA expression levels of three of the major circadian clock genes. In parallel, we compared the results obtained here with the locomotor/flight activity described for *Ae. aegypti* male mosquitos. Our results suggest that at least one gene, *cry2*, may be a player involved in a regulatory pathway associated with pre-copulation social interaction. We believe that further functional studies of *cry2*, focusing on social interaction driven to reproduction, could help elucidate whether this gene has a role in *Ae. aegypti*'s reproductive success.

## Supplementary Information

**Additional file 1: Table S1**. Means and standard errors of the clock genes in tissues of *Aedes aegypti* males

**Additional file 2: Table S2**. Sheets of raw gene expression data for the three genes in heads of males with antennae, in all conditions. Data of three experimental replicates are included.

**Additional file 3: Table S3**. Sheets of raw gene expression data for the three genes in heads of males without antennae, in all conditions. Data of three experimental replicates are included.

**Additional file 4: Table S4**. Sheets of raw gene expression data for the three genes in abdominal tips of males with antennae, in all conditions. Data of three experimental replicates are included.

**Additional file 5: Table S5**. Sheets of raw gene expression data for the three genes in abdominal tips of males without antennae, in all conditions. Data of three experimental replicates are included.

**Additional file 6: Table S6**. Sheets of raw gene expression data for the three genes in antennae of males, in all conditions. Data of three experimental replicates are included.

## Data Availability

Conclusions in this article are supported by the information available in the article. The datasets used and analyzed during the study are available from the corresponding author upon request.
